
JOURNAL INFORMATION
==============================
NLM Title Abbreviation: South Med Rec
NLM Journal ID: 0027317

The Southern Medical Record

Publisher: Biomedical Journal Digitization Project

ARTICLE INFORMATION
==============================
PMCID: PMC9089503
PMID: 36023115
Article version: 1
Subjects: Editorial and Miscellaneous

Medical Association of Georgia

Print publication date: 1879 May 20
Volume: 9
Issue: 5
First page: 200
Last page: 200
License: This work is free of known copyright restrictions. For more information, please see PMC Back Issue Digitization.
License URL: https://creativecommons.org/publicdomain/mark/1.0/

==============================

### *MEDICAL ASSOCIATION OF GEORGIA.*

The Medical Association of Georgia at Rome, on 16th ult. was well attended. The hospitality extended to members by the Profession and citizens was warm and liberal. The following are the officers elected for the ensuing year:

| | |
|---|---|
| Dr. Jos. A. Eve, President. | Dr. K. P. Moore, Treasurer. |
| Dr. J. C. LeHardy, 1st Vice President. | Dr. W. S, Kendrick, Orator. |
| Dr. B. R. Dostor, 2d Vice President. | Dr. W. B. Wells, } Censors. |
| Dr. James B. Baird, Secretary. | Dr. Jno. S. Coleman, } Censors. |

*Committee on Publication.*

Jas. B. Baird, ex officio; K. P. Moore: W. S. Armstrong; V. H. Taliaferro; Wm. Abram Love.

*Committee on Necrology.*

R. P. Myers; H. V. Johnson; W. H. Hall; W. A. Love; A. Sibley Campbell.

*Committee on State Board of Health.*

Jas. B. Baird; W. S. Armstrong; C. A. Simpson; W. A. Love; J. C. LeHardy.

*Delegates to the American Medical Association, Atlanta, May 6th*, 1879.

| | |
|---|---|
| Wm O Daniel, | W T Goldsmith, |
| J C LeHardy, | P H Wright, |
| J P Logan, | E L Connally, |
| H F Campbell, | T J Word, |
| Robt. Battey, | G F Cooper, |
| T S Hopkins, | W D Hoyt, |
| V H Taliaferro, | L A Dugas, |
| W F Holt, | J G Westmoreland, |
| A P Taylor, | J G Thomas, |
| J T Banks, | F A Stanford, |
| A W Calhoun, | T H Keenan, |
| E J Roach, | J T Johnson. |
| J B S Holmes, | |

A Resolution was passed inviting all who have been dropped from the roll for non-payment of dues to reunite with the Organization upon the payment to the Secretary of $2.00 at any time during the present year.

### *ELECTION OF FACULTY.*

We are authorized to state that a meeting of the Board of Trustees of the Southern Medical College will be held on the 16th of June, for the purpose of electing Professors to the several Chairs of the Institution.

Notice of *Convention of Colleges*, *Book Notices* and *Subscribers Receipted* are crowded out and will appear in next issue.

### SPECIAL NOTICES.

**Sacramental Wine.** Speer's Port Grape Wine is a pure, unintoxicating Wine, from the finest native grown Port Grapes, especially for the use of Christian Churches, and guaranteed to retain its grateful flavor and essential qualities unimpaired for any period. Much used for evening parties and by invalids. For sale by druggists.

**Messrs. Parke, Davis & Co.**, of Detroit, Mich., have for the past few years enjoyed the reputation of being one of the most enterprising firms in America, in introducing new remedies and disseminating useful information regarding them. They have their own agents in Mexico, South America, Great Britain, and on the Continent, who are constantly seeking out new remedies, and procuring facts regarding their medical value. Their list of manufactures comprises: Fluid and Solid Extracts, Sugar-Coated Pills, Medical Lozenges, Medicated Syrups, Elixirs, Wines and Syrups, Medicated Collodions, Confections, Cerates, Effervescent Salts, Fumigating Pastilles, Pepsin, Pancreatine, Ergotine, Aqua Ammoniæ, Chem. Pure Chloroform, Empty Capsules, Chlor-Anodyne, etc., etc.—*New Remedies.*

PARIS, February 20th, 1879.

*Editors North Carolina Medical Journal.*

GENTLEMEN: * * * * * A Medicine has recently been introduced into France by our enterprising countrymen, **Messrs. Wm. R Warner & Co.**, of Philadelphia *INGLUVIN* (a powder prescribed in the same manner and dose as Pepsin), prepared from the gizzard of the chicken. I am pleased to be able to chronicle the fact, that, in three cases of pronounced atonic dyspepsia and in one case of chronic indigestion, it has acted like a charm—promptly relieving all disagreeable symptoms, and restoring the stomach to its proper functions. My patients, who had previously tried without benefit all ordinary forms of pepsine, bismuth, cerium, nux vomica, etc., etc., are delighted with this new remedy, and assure me that they experienced benefit from the first dose. Hereafter I shall prescribe it liberally and with great confidence in its therapeutic value. Very truly and respectfully yours,

EDWARD WARREN, (BEY) M.D., C. M.
